# Bioprocess optimization of *Metschnikowia pulcherrima*: carbon source selection, nutrient ratio tuning, and environmental parameter modeling using response surface methodology

**DOI:** 10.1016/j.btre.2026.e00984

**Published:** 2026-10-10

**Authors:** Bogdan T. Dinić, Nicholas A. Bokulich, Florian M. Freimoser, Ueli von Ah

**Affiliations:** a Agroscope, Department of Food Microbial Systems, Posieux, 1725, Switzerland; b ETH Zürich, Institute of Food, Nutrition, and Health, Department of Health Sciences and Technology, Zurich, 8092, Switzerland; c Agroscope, Department of Plant Protection, Nyon, 1260, Switzerland

**Keywords:** *Metschnikowia pulcherrima*, Bioprocess optimization, CCD, Carbon-nitrogen-ratio, Crabtree effect

## Abstract

Although *Metschnikowia pulcherrima* shows promise as a biocontrol agent, its upstream development remains limited. We maximized the viable cell count of *M. pulcherrima* strain APC 1.2 by employing a Central Composite Design (CCD) to optimize pH and temperature. Preliminary screening identified sucrose at 20 g/L and 1600 mg YAN/L yeast extract as optimal for viable cell count (CFU/mL), with further increases in YAN providing no additional benefit. The highest viable cell count was achieved at milder temperatures (18–22 °C), where the strain grew robustly across a wide pH range (3.59–6.41), while higher temperatures increased sensitivity to acidic pH and reduced viable cell count. The strain was confirmed to be Crabtree-negative, showing no ethanol production during aerobic growth on glucose. The proposed workflow enabled reproducible optimization of cultivation parameters, providing a scalable framework for industrial applications, and laying the groundwork for future studies on non-conventional yeasts in biocontrol and industrial biotechnology.

## Highlights

• Best tested medium was found to be 20 g/L sucrose and 1600 mg YAN/L yeast extract.
• *Metschnikowia pulcherrima APC 1.2* confirmed Crabtree-negative under aerobic conditions.
• Response surface methodology identified optimal pH at 4.57 and temperature at 17.95 °C.
• Model predictions matched observations at 500 mL and 6 L bioreactor working volumes.

## 1 Introduction

Chemical pesticides have long been used to manage plant diseases, but concerns about environmental impact, human health, and pathogen resistance have highlighted the need for safer alternatives. Biological control, which uses living organisms to suppress plant pathogens, offers a sustainable approach to crop protection [1]. The biocontrol agent market is experiencing double-digit growth and is expected to grow from USD 5 billion to USD 15 billion by 2029 [2]. Several yeast species show considerable potential as biocontrol agents against plant-pathogenic fungi and can serve as eco-friendly alternatives to chemical pesticides [3]. One of the most promising biocontrol species is the non-*Saccharomyces* yeast *Metschnikowia pulcherrima*, which exhibits strong antagonistic activity against a range of plant pathogens, particularly those responsible for post-harvest rot [4]. Certain strains have demonstrated efficacy against *Botrytis cinerea, Penicillium expansum*, and *Alternaria alternata* among others, and their performance has been extensively studied on various fruits and vegetables, including apples [[5], [6], [7], [8], [9]], grapes [10] and grape juice/must [11,12], citrus fruits [[13], [14], [15]], and potatoes [16] to name a few.

Previously, Hilber-Bodmer et al. [4] found that *M. pulcherrima* strain APC 1.2, isolated from apple phyllosphere, was the most strongly antagonistic yeast strain (among 40 different yeasts tested) against 16 filamentous fungi. Additionally, this same strain demonstrated a strong competitive ability against other antifungal yeasts on apples [17]. The antagonistic activity of *M. pulcherrima* is thought to be mainly due to the production of the iron chelator pulcherriminic acid [18]. The role of pulcherriminic acid in mediating biocontrol activity has been shown using a natural *M. pulcherrima* mutant, but other factors must also contribute to the strong antagonistic effects [19].

While *M. pulcherrima* has demonstrated promise as a biocontrol agent, the upstream development for this application remains limited. To date, only a few studies focused on optimizing upstream production of *M. pulcherrima*, with the primary focus being on lipid or biodiesel production [[20], [21], [22], [23], [24], [25], [26]]; or oenology [27]. Research on production for biocontrol agents is sparse, particularly regarding viable cell count (VCC) optimization [28,29], a key factor for biocontrol efficacy, as the living cells of *M. pulcherrima* are required for its biocontrol abilities [8,16]. Spadaro et al. [29], investigated the effect of pH on growth using shake flasks at a constant temperature, providing initial insights into this parameter. However, the combined effects of pH and temperature have not been systematically explored under controlled cultivation conditions. Using a statistical approach such as Central Composite Design (CCD) makes it possible to evaluate several factors simultaneously and understand how they interact, rather than testing them in isolation. As part of the broader Response Surface Methodology (RSM), CCD is widely applied because it can capture both linear and non-linear effects, making it a powerful tool for process optimization. This not only improves the accuracy of the results but also supports the development of reproducible and scalable cultivation processes while reducing the number of experimental runs [[30], [31], [32], [33]].

A crucial metabolic characteristic to consider for growth optimization is whether a yeast species exhibits the Crabtree effect, i.e., fermentation in the presence of oxygen under certain growth conditions. Previous studies have indicated that *M. pulcherrima* exhibits Crabtree-negative behavior when grown in molasses-based medium, a complex mixture containing mainly sucrose [27,34,35]. Conversely, Spadaro et al. [29] attributed the absence of an increase in VCC when increasing yeast extract from 40 g/L to 60 g/L to the Crabtree-positive metabolism, although ethanol production was not measured. However, mixed carbon sources and enzymatic hydrolysis complicate the interpretation of metabolic traits. Unlike *K. lactis*, which does not produce ethanol under aerobic conditions (Crabtree-negative), Crabtree-positive *S. cerevisiae* strains begin aerobic ethanol production at relatively low glucose concentrations [36]. The precise threshold for the Crabtree effect can be species-dependent [37].

In this study, we aimed to investigate the optimal nitrogen-to-carbon source ratio, temperature, and pH conditions to maximize the viable cell count of *M. pulcherrima* strain APC 1.2 by using a Central Composite Design (CCD), with the goal of improving cost-efficiency and sustainability in bioprocess design. To evaluate the Crabtree metabolism of the strain, we designed a controlled experiment using glucose as the sole carbon source, which is central to Crabtree metabolism, and included both a Crabtree-positive control *Saccharomyces cerevisiae* [36] and a Crabtree-negative control *Kluyveromyces lactis* [38]. This approach allows for a more precise, reproducible, and scalable optimization of cultivation conditions tailored for biocontrol applications. The results of this study will help advance the production of high-performance microbial biocontrol products and highlight the benefits of using data-driven approaches in industrial microbiology.

## 2 Materials and methods

### 2.1 Microorganism and inoculum preparation

Three yeast strains were used in this study: *Metschnikowia pulcherrima* APC 1.2 ([4]; CCoS978) obtained from Agroscope Changins, *Saccharomyces cerevisiae* ATCC 201388 was obtained from the American Type Culture Collection (ATCC, Manassas, Virgina, USA), and *Kluyveromyces lactis* FAM-27490, retrieved from the Agroscope Liebefeld strain collection. *K. lactis* was scraped from the master culture and inoculated into 10 mL of a custom medium (30 *g*/L Trypticase Soy Broth, BBL 211,768, BD Diagnostics; 5 *g*/L d-(+)-Glucose, Merck 8342; 5 *g*/L Yeast Extract, BBL 211,929, BD Diagnostics) and incubated for 48 h at 30 °C. This culture was then poured into a 250 mL DURAN® baffled Erlenmeyer flask (DWK Life Sciences, Germany) with an additional 40 mL in a liquid medium optimized for *M. pulcherrima*, as described by Spadaro et al. [29], consisting of 7.5 g/L d-mannitol and 30 g/L yeast extract. Among the single carbon sources tested, D-mannitol (7.5 g/L) supported the highest viable cell count (1.5 × 10⁹ CFU m/L), comparable to those obtained with a mannitol-sorbose mixture (5 g/L each; 1.7 × 10⁹ CFU m/L). From this culture, 10 mL was transferred into 90 mL of the same medium in a 500 mL DURAN® baffled Erlenmeyer flask (DWK Life Sciences, Germany) and incubated at 25 °C with shaking at 170 rpm for 24 h. The same two-step propagation procedure was applied to *M. pulcherrima* and *S. cerevisiae*, starting directly from potato dextrose agar plates (Potato Dextrose Agar, Sigma-Aldrich P2182; agar 15 g/L, dextrose 20 g/L, potato infusion 4 g/L). After the second incubation, all cultures were concentrated twofold and preserved in reconstituted skim milk at −40 °C for later use. This protocol ensured consistent cell viability and reproducibility across experiments.

### 2.2 Sugar screening in shake flasks

The base medium consisted of 30 g/L yeast extract (NUCEL® 545 MG, Procelys by Lesaffre, France), and the initial pH was adjusted to 5.0 using 1 N hydrochloric acid (HCl 6 N, Grogg Chemie, Germany), except for two treatments - 20 g/L sucrose and 7.5 g/L d-mannitol - where the pH was not adjusted.

Carbon sources included monosaccharides (D-glucose (≥99.5%, Tereos Starch & Sweeteners Belgium NV, Belgium), d-fructose (≥99%, ACS Reagent, CAS 57–48–7, Sigma-Aldrich, USA)), disaccharides (sucrose (≥97%, Schweizer Zucker AG, Switzerland), maltose (≥97%, CAS 6363–53–7, Sigma-Aldrich, USA)), and a polyol d-mannitol (≥99%, CAS 69–65–8, Sigma-Aldrich, USA). Concentrations ranged from 5 to 30 g/L, with selected combinations designed to assess potential synergistic effects. One condition included a mixture of glucose and fructose (10.5 g/L each, ≈0.058 mol/L each), were combined in a total molar amount equivalent to that produced by hydrolyzing 20 g/L sucrose (≈0.058 mol/L) Table 1.

**Table 1:** Carbon sources and concentrations tested in this study. Monosaccharides (glucose, fructose), disaccharides (sucrose, maltose), along with d-mannitol (sugar alcohol) and selected combinations were evaluated. Conditions marked with an asterisk (*) indicate experiments without pH adjustment.

#	D-Mannitol (g/L)	Glucose (g/L)	Fructose (g/L)	Sucrose (g/L)	Maltose (g/L)
1	-	-	-	-	-
2	5	-	-	-	-
3	7.5	-	-	-	-
4	7.5*	-	-	-	-
5	12.5	-	-	-	-
6	-	10	-	-	-
7	-	20	-	-	-
8	-	-	10	-	-
9	-	-	20	-	-
10	-	-	-	10	-
11	-	-	-	20	-
12	-	-	-	20*	-
13	-	-	-	25	-
14	-	-	-	30	-
15	-	-	-	-	10
16	-	-	-	-	15
17	5	10	-	-	-
18	5	-	-	10	-
19	-	10.5	10.5	-	-
20	-	-	-	10	10

All conditions were tested in biological triplicates. Cultures were grown in 500 mL DURAN® baffled Erlenmeyer flask (DWK Life Sciences, Germany) containing 100 mL of medium and incubated at 25 °C with agitation at 170 rpm for 48 h in an orbital shaker. For each sample, 10-fold serial dilutions of the culture suspensions were prepared in duplicate in sterile 48-well cell culture plates using 0.9% w/v saline solution (≥99.8%, JuraSel® Speisesalz, Schweizer Salinen AG, Switzerland) and a multichannel pipette with adjustable spacing. Diluted samples were plated on Difco™ Plate Count Agar (PCA; BD Diagnostics) using a spiral plate system (Eddy Jet 2 W, IUL S.A, Spain). Plates were incubated at 30 °C for 2–3 days, and colony counts were obtained using the SphereFlash® Automatic Colony Counter (IUL S.A, Spain).

### 2.3 Sugar to yeast extract ratio optimization in shake flasks

Three sucrose concentrations (20, 35, and 50 g/L) were tested in combination with five YAN levels (100, 600, 1100, 1600, and 2100 mg YAN/L), as shown in Table 2. Yeast extract (NUCEL® 545 MG, Procelys by Lesaffre, France) was used as the nitrogen source, with 1 g/L of yeast extract corresponding to approximately 52.5 mg YAN/L. Yeast assimilable nitrogen is the total nitrogen content available for yeast metabolism. It is expressed in milligrams of nitrogen per liter (mg N/L) and includes ammonium ions (NH₄⁺) and free amino acids, excluding proline and hydroxyproline due to their limited uptake by yeast [39]. In this study, YAN was quantified using a combination of enzymatic and colorimetric methods by using Biosystems S.A. kits (Barcelona, Spain). Ammonium was measured enzymatically, while amino nitrogen was determined calorimetrically. All conditions were tested in biological triplicates. Cultivation, plating, and CFU quantification were performed as described in Section 2.2.

**Table 2:** Mixed full factorial design of sucrose and yeast extract concentrations. Condition numbers C1-C15 represent 15 unique mixtures combining three sucrose concentrations (20, 35, and 50 g/L) with five yeast extract concentrations (100, 600, 1100, 1600, and 2100 mg YAN/L).

Yeast extract (mg YAN/L)	Sucrose 20 g/L	Sucrose 35 g/L	Sucrose 50 g/L
100	C1	C2	C3
600	C4	C5	C6
1100	C7	C8	C9
1600	C10	C11	C12
2100	C13	C14	C15

### 2.4 Bioreactor setup and conditions

All bioreactor-based experiments were conducted using Gettinge Applikon MiniBio 1000 mL stirred-tank bioreactors (Getinge AB, Sweden) with a working volume of 500 mL. Each unit was equipped with sensors for pH (AppliSens pH Sensor, Getinge AB, Sweden), dissolved oxygen (DO) (AppliSens Optical DO Sensor, Getinge AB, Sweden), temperature, and agitation. The bioreactors were operated via Lucullus® PIMS 24.1.1 (Securecell AG, Switzerland) and controlled using the Applikon My-Control unit (Getinge AB, Sweden). Bioreactors were filled with 500 mL of the respective culture medium and sterilized in an autoclave at 121 °C for 15 min. Prior to sterilization, the vessels were connected to the control system and the probes calibrated according to the manufacturer’s instructions. Aeration was provided using filtered air at a maximum flow rate of 2 vvm. The target DO level was set at 100%, and it was managed using a cascade control strategy, adjusting agitation speed between 250 and 1250 rpm. The pH was automatically maintained at 5.0 using 1 N NaOH (Emsure® NaOH, Merck, Germany). Antifoam (Antifoam Struktol J 673 A, Schill & Seilacher, Germany) was added manually or automatically as needed. Sampling was performed aseptically through a dedicated port using sterile syringes. Pre-cultures were prepared by inoculating 1% (v/v) of working stock into 100 mL of the trial medium in a 500 mL DURAN® baffled Erlenmeyer flask (DWK Life Sciences, Germany) and incubated at 25 °C with shaking at 170 rpm for 24 h. Bioreactors were inoculated with 1% (v/v) of the pre-culture.

This setup was used consistently across all bioreactor experiments described in 2.5, 2.6.

For the additional model validation, experiments with a 6.0 L working volume were conducted in 10 L bioreactors (Bilfinger Industrietechnik, Salzburg, Austria), each integrated with the Lucullus® process control console from the advanced biocontrol system (Securecell AG, Switzerland). The bioreactors were equipped with a dissolved oxygen (DO) sensor (OxyFerm®, Hamilton, Switzerland) and a pH sensor (EasyFerm® Bio, Hamilton, Switzerland), both designed for hygienic bioprocess applications. Antifoam (Antifoam Struktol J 673 A, Schill & Seilacher, Germany) was added manually or automatically as needed. Operating conditions were maintained as follows: temperature was controlled at 20 °C; pH was maintained at 6.0 and regulated automatically using 1 N NaOH; dissolved oxygen was set at 100% saturation; agitation varied between 200 and 1200 rpm to ensure optimal mixing and oxygen transfer; and aeration was provided via a submerged ring sparger, with airflow regulated between 0 - 1.5 vvm using cascade regulation. For each sample, cultivation, plating, and viable cell count evaluation was performed as described in Section 2.2.

### 2.5 Crabtree effect trials

The Crabtree effect in *M. pulcherrima* was evaluated in comparison with a known Crabtree-positive yeast (*Saccharomyces cerevisiae* ATCC 201,388) and a Crabtree-negative control (Kluyveromyces *lactis* FAM-27,490). Experiments were conducted in Gettinge Applikon MiniBio 1000 mL stirred-tank bioreactors (Getinge AB, Sweden) with a working volume of 500 mL, as described in Section 2.4 at 30 °C for 48 h. The bioreactors were filled with 500 mL of culture medium, consisting of 20 g/L d-glucose and 30 g/L yeast extract, and subsequently sterilized by autoclaving at 121 °C for 15 min. All experiments were conducted in duplicates.

Samples (5 mL) were collected at 0, 4, and 8 h, then every 2 h from 20 to 32 h, and finally at 48 h. CFU/mL was determined using the plating method described in Section 2.2. Glucose consumption and ethanol production were quantified using high-performance liquid chromatography with an Agilent 1260 Infinity HPLC system (Agilent Technologies, USA) connected to the Securecell Numera® automated sampling platform for sample preparation and transfer. Separation was achieved on an Aminex HPX-87H Ion Exclusion Column (300 × 7.8 mm; Bio-Rad, USA), operated at 55 °C. The mobile phase consisted of 10 mM H₂SO₄ prepared by dilution from a 0.5 mol/L stock solution (Merck, Art. 1.01040) at a flow rate of 0.65 mL/min, and detection was performed using a refractive index (RI) detector. An injection volume of 20 µL was used.

### 2.6 Central composite design for pH and temperature

The effects of pH and temperature on microbial viable cell count were evaluated using a Central Composite Design (CCD). All experiments were performed in 1 L stirred-tank bioreactors, as described in Section 2.4, under consistent control of pH, temperature, dissolved oxygen, and agitation to ensure process reproducibility. The bioreactors were filled with 500 mL of culture medium, consisting of 20 g/L sucrose (≥97%, Schweizer Zucker AG, Switzerland) and 1600 mg YAN/L (approximately 30 g/L) of yeast extract (NUCEL® 545 MG, Procelys by Lesaffre, France), and subsequently sterilized by autoclaving at 121 °C for 15 min. All experiments were conducted in duplicates. Samples were taken every 4 h from 24 h to 36 h, and for the modelling the first point was taken after 28 h which was previously determined as start of the stationary phase for all conditions.

Two independent variables were investigated: pH (X_pH_) and temperature (X_temp_), each evaluated at five coded levels: –α, –1, 0, +1, and +α. The axial distance (α) was set to 1.41 to achieve rotatability. A 2² Central Composite Circumscribed (CCC) design was constructed, comprising 12 experimental runs: four factorial points, four axial points, and four center points. This design enables efficient estimation of second-order effects with a minimal number of runs, offering good predictive capability near the optimum [40].

To preserve orthogonality and reduce potential block-related variability, the experimental runs were distributed across two blocks: Block 1 included the factorial points and two center runs, while Block 2 comprised the axial points and the remaining two center runs. The runs were randomized within each block.

The coded regression model for the response (viable cell count) is expressed as (Eq. (1)):

(1) $$Y=\beta_{0}+\beta_{1}X_{pH}+\beta_{2}X_{\text{temp}\;}+\beta_{12}X_{pH}X_{\text{temp}\;}+\beta_{11}{X_{pH}}^{2}+\beta_{22}{X_{\text{temp}\;}}^{2}$$

Where Y is the predicted response variable of the viable cell count, Xₚ_H_ and Xₜₑₘₚ are the coded values of pH and temperature; β₀ is the intercept; β₁ and β₂ represent the linear effects of pH and temperature, respectively; β₁₁ and β₂₂ are the quadratic effects of pH and temperature; and β₁₂ represents the interaction effect between pH and temperature.

Test variables were coded for statistical analysis and regression according to (Eq. (2)):

(2) $$X_{i}=\frac{\left(Z_{i}-Z_{0}\right)}{\Delta Z_{i}}$$

In this equation, X*_i_* represents the coded form of the independent variable, Z*_i_* is its actual measured value, Z*_0_* marks the value at the central point of the design, and ΔZ*_i_* is the increment corresponding to one unit of the coded variable.

The actual values corresponding to the coded levels were as follows Table 3:

**Table 3:** Central composite design (CCD) with coded and actual values of pH and temperature. Coded levels (−α, −1, 0, +1, +α) correspond to the experimental ranges of pH and temperature used in this study. The center point (0) represents the midpoint of the tested range for each factor, while ±1 indicates the factorial levels and ±α the axial points. Actual pH and temperature values are provided to allow direct interpretation of experimental conditions.

Coded levels	–α	−1	0	+1	+ α
pH	3.59	4.0	5.0	6.0	6.41
Temperature ( °C)	17.95	20.0	25.0	30.0	32.05

Therefore, the following equations were used to convert coded values to actual pH (Eq. (3)) and temperature (Eq. (4)) levels:

(3) $$X_{pH}=pH-5$$

(4) $$X_{temp}=\frac{\left(T-25\right)}{5}$$

Where (X_pH_) and X_temp_), represent the coded values corresponding to pH and temperature, pH is the measured pH of the medium, and T is the actual temperature of the medium (°C).

This design enabled the estimation of linear effects, interactions, and quadratic effects of the variables, and supported the development of a second-order polynomial model. The full experimental matrix is presented in Table 4.

**Table 4:** Central Composite Design (CCD) Experimental Design Matrix showing run number, block, and the coded and actual values of pH and temperature.

Run Number	Block	Coded pH (X₁)	Coded Temperature (X₂)	Actual pH	Actual Temperature ( °C)
1	1	−1.0	−1.0	4.0	20.0
2	1	1.0	−1.0	6.0	20.0
3	1	−1.0	1.0	4.0	30.0
4	1	1.0	1.0	6.0	30.0
5	1	0.0	0.0	5.0	25.0
6	1	0.0	0.0	5.0	25.0
7	2	−1.41	0.0	3.59	25.0
8	2	1.41	0.0	6.41	25.0
9	2	0.0	−1.41	5.0	17.95
10	2	0.0	1.41	5.0	32.05
11	2	0.0	0.0	5.0	25.0
12	2	0.0	0.0	5.0	25.0

### 2.7 Statistical analysis

For carbon source screening and Crabtree effect trials, GraphPad Prism (version 10.2.3 (403), GraphPad Software, San Diego, CA, USA) was used for data visualization and statistical testing. Data is presented as mean ± standard deviation.

To compare multiple carbon sources, one-way analysis of variance (ANOVA) followed by Tukey’s honestly significant difference (HSD) post hoc test was employed to determine statistically significant differences among groups (p < 0.05).

In sugar to yeast extract ratio optimization GraphPad Prism was used for data visualization, while JMP® software (version (Student Edition) 18.2.2, SAS Institute Inc., Cary, NC, USA) was used for statistical analysis and model fitting. Statistical analysis, model fitting, ANOVA, and graphical representation for the Central Composite Design (CCD) was performed using JMP® software. The design parameters, including the axial distance (α) and model fitting criteria, were calculated following the guidelines described by Box et al. [40].

All tests assumed a level of significance α = 0.05.

## 3 Results

### 3.1 Sugar screening in shake flasks

An initial screening was conducted to evaluate the effect of various carbon sources and their combinations on viable cell count (VCC). A total of 20 carbon source conditions, including various sugar types, mixtures, and two non-pH-adjusted treatments, were evaluated after 48 h.

Overall, a one-way ANOVA revealed a statistically significant effect of the treatments on the viable cell counts (**F(19, 32) = 39.83, *p*** **<** **0.0001**), with **R² = 0.9594**, confirming that 95.9% of the variability in cell counts was attributable to treatment differences. Maximum VCC was observed at 20–30 g/L sucrose, with a significant rise from 10 to 20 g/L and no further gains at higher concentrations (Fig. 1). Maltose and sucrose, at a concentration of 10 g/L, resulted in comparable VCC, but increasing maltose to 15 g/L did not enhance VCC. Only when combined with sucrose (10 g/L each) did the VCC approach the maximum observed with high sucrose concentrations, suggesting a potential synergistic effect. Disaccharides outperformed monosaccharides, and a glucose-fructose mixture (10.5 g/L each, ≈0.058 mol/L each), equimolar to 20 g/L sucrose (≈0.058 mol/L), used to simulate hydrolysis, resulting in significantly lower growth compared to sucrose. A similar trend was observed when comparing glucose (10 g/L and 20 g/L) with maltose (10 g/L); despite maltose having a slightly lower carbon content, it supported significantly higher VCC. Growth on d-mannitol resulted in lower VCC, comparable to glucose and fructose. Combining mannitol with either glucose or sucrose did not lead to a synergistic increase in VCC and yielded similar or lower results than glucose or sucrose alone. Non-pH-adjusted media (pH ≈ 6.83) significantly altered the results: 20 g/L sucrose supported lower, whereas 7.5 g/L d-mannitol higher VCC compared to their pH-adjusted counterparts (initial pH = 5), highlighting the importance of pH control.

**Fig. 1:**
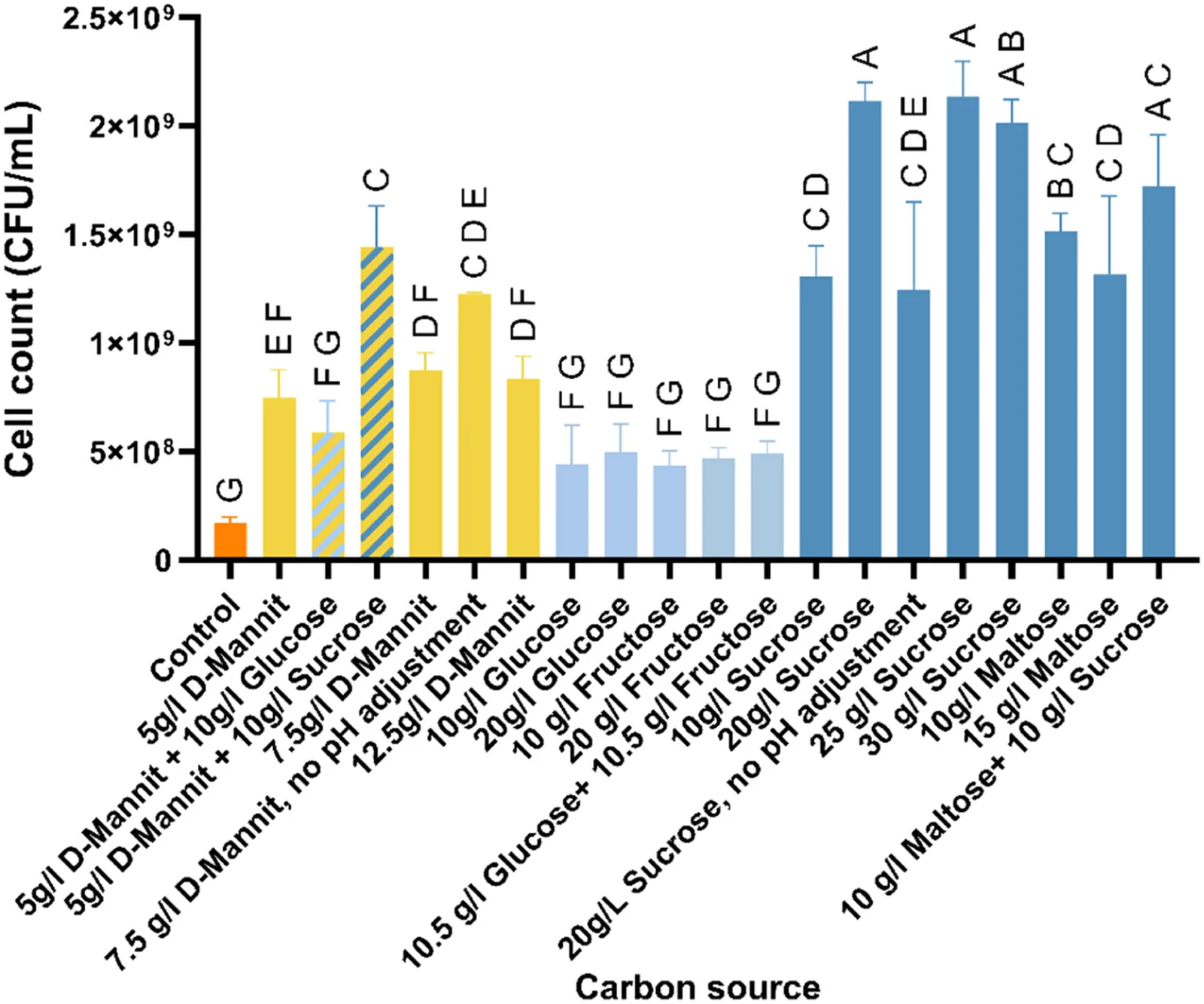
Comparison of mediums with different carbon sources and 30 g/L Yeast Extract. Unless otherwise stated the initial pH was set to pH 5.0 (25 °C, 170 rpm, 48 h). Different letters above bars indicate statistically significant differences (Tukey’s HSD, p < 0.05). Identical letters denote no significant difference.

### 3.2 Optimization of sugar to yeast extract ratio in shake flasks

To refine the carbon-to-nitrogen balance for optimal microbial growth, a mixed full factorial design was implemented, focusing on sucrose and yeast extract concentrations. The aim was to determine the optimal sugar-to-nitrogen ratio, with nitrogen availability expressed as yeast assimilable nitrogen (YAN). A mixed full factorial design with 3 sucrose levels (20, 35 and 50 g/L) and 5 yeast extract levels (100, 600, 1100, 1600, 2100 mg YAN/L) was implemented to investigate the impact of sucrose and yeast extract.

A second-order regression model was fitted to the log₁₀-transformed viable cell counts (VCC) of the individual flasks (mixed full factorial dataset; see Supplementary 1). The fitted model indicated a good fit of the observed data, with a coefficient of determination (R²) of 0.89 and an adjusted R² of 0.87, indicating that the model explained a significant portion of the variability despite the biological complexity of the system. The model confirmed a significant main effect of yeast extract concentration (p < 0.0001), including significant curvature (quadratic term, p < 0.0001) consistent with an optimum, while sucrose concentration in the tested range did not influence viable cell count (p = 0.47).

A heat map of the viable cell count data (Fig. 2) visually reinforces this interpretation, where a clear trend was observed in response to increasing yeast extract concentrations. Across all sugar levels, higher yeast extract levels generally resulted in increased VCC, with a noticeable peak being reached at 1600 mg YAN/L. However, beyond this level (at 2100 mg YAN/L approximately 40 g/L yeast extract), VCC did not improve significantly with 20 g/L sucrose and decreased with 35 g/L sucrose and 50 g/L sucrose. This indicated that, under our experimental conditions, there is a threshold for yeast extract supplementation below which cell VCC is compromised, highlighting the importance of optimizing nitrogen availability in relation to carbon source concentration.

**Fig. 2:**
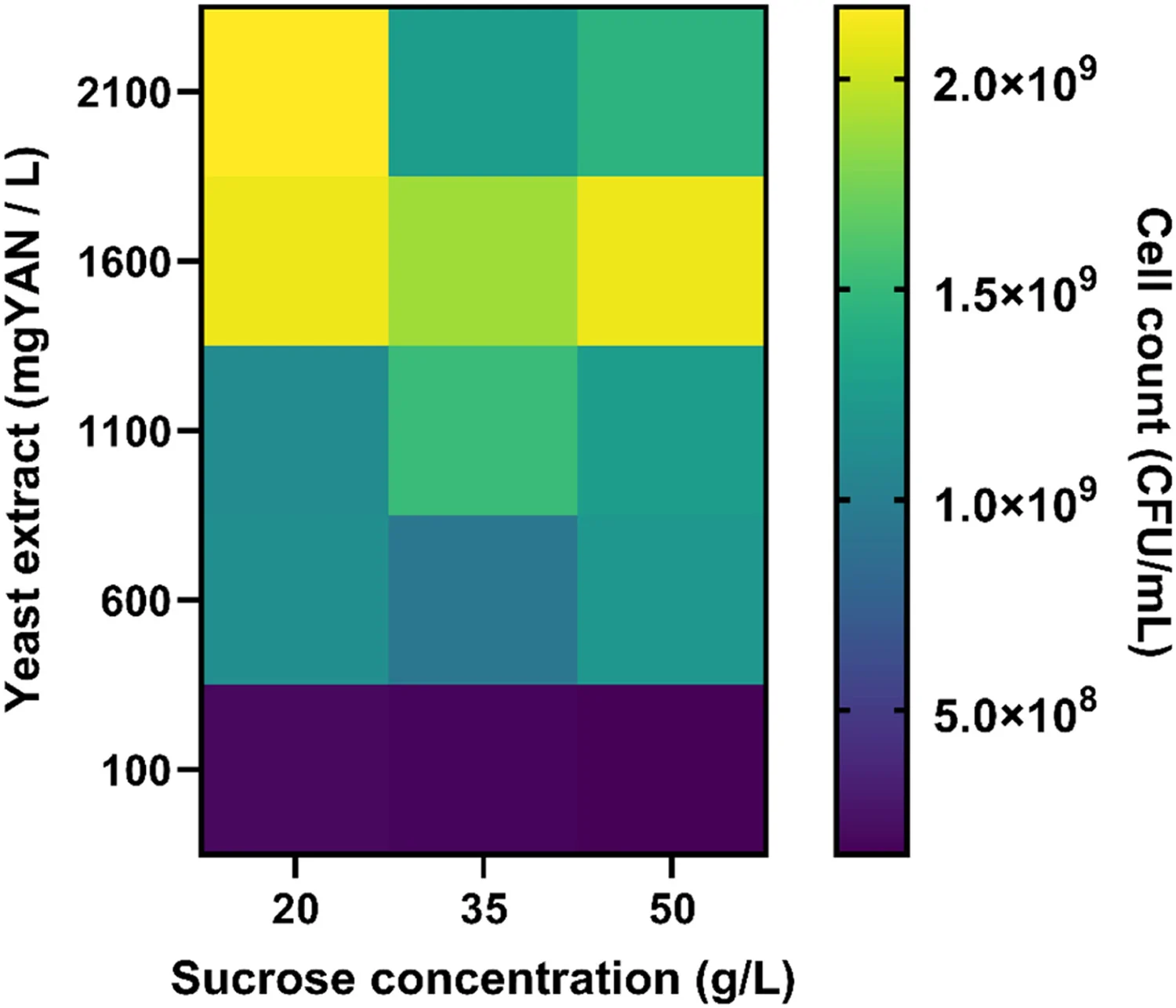
Heat map of CFU counts across full factorial nutrient conditions. Rows represent increasing yeast extract concentrations (100 –2100 mg YAN/L), columns represent sucrose levels (20 – 50 g/L). The strongest growth signal is concentrated at the intersection of 1600 mg YAN/L Yeast Extract and 20 g/L Sucrose.

The highest number of viable cells was obtained with 20 g/L sucrose and either 1600 or 2100 mg YAN/L and with 50 g/L sucrose and 1600 mg YAN/L. Given that three conditions gave comparable results, the formulation with the lowest concentrations of sucrose and yeast extract was selected to reduce resource consumption. Thus, the formulation containing 20 g/L sucrose and 1600 mg YAN/L yeast extract was selected for pH and temperature optimization in the subsequent Central Composite Design (CCD) trials. To validate the robustness of the identified optimal ratio, two additional conditions were tested: one with a fivefold increase in sugar concentration (100 g/L sucrose, 1600 mg YAN/L ≈ 30 g/L yeast extract), and another with a fivefold increase in both sugar and yeast extract (100 g/L Sucrose, 150 g/L yeast extract). In both cases, the observed responses were similar or lower (2.05×10^9^ CFU/mL and 1.31×10^9^ CFU/mL, respectively) than those at the optimal mixture, with the stationary phase being reached after three days, compared to two days for other samples. These findings suggested that increasing concentrations beyond the optimal range does not improve performance and may even reduce VCC in shaking flasks.

### 3.3 Evaluation of the Crabtree effect

To accurately assess the Crabtree metabolism of *M. pulcherrima*, ethanol production was monitored under aerobic conditions to isolate intrinsic metabolic behavior. This approach provides a clearer framework for characterizing the Crabtree effect in *M. pulcherrima* and aims to strengthen the reproducibility and interpretability of previous findings. Crabtree metabolism of *M. pulcherrima* was compared with a Crabtree positive control (*S. cerevisiae*) [36] and Crabtree negative control (*K. lactis*) [38]. Experiments were conducted in 1 L Applikon bioreactors with dissolved oxygen (DO) set at 100%, as described in Section 2.4. Under these aerobic conditions, no ethanol production was observed, supporting the conclusion that the *M. pulcherrima* isolate APC 1.2 is a Crabtree-negative yeast (Fig. 3). This was also confirmed at higher concentrations of glucose (30 g/L) and sucrose (100 g/L). Unlike *K. lactis*, which showed no ethanol production, Crabtree positive *S. cerevisiae*, which is known to exhibit this effect in certain strains at concentrations as low as 150 mg/L glucose [36] produced ethanol under aerobic conditions until the end of exponential phase, after which the ethanol concentration decreased from 8.15 g/L after 24 h to 4.70 g/L after 48 h. The observed reduction in ethanol concentration may be attributed either to consumption of ethanol by *S. cerevisiae* during the diauxic shift [41,42] or to physical loss from the bioreactor medium due to gas stripping.

**Fig. 3:**
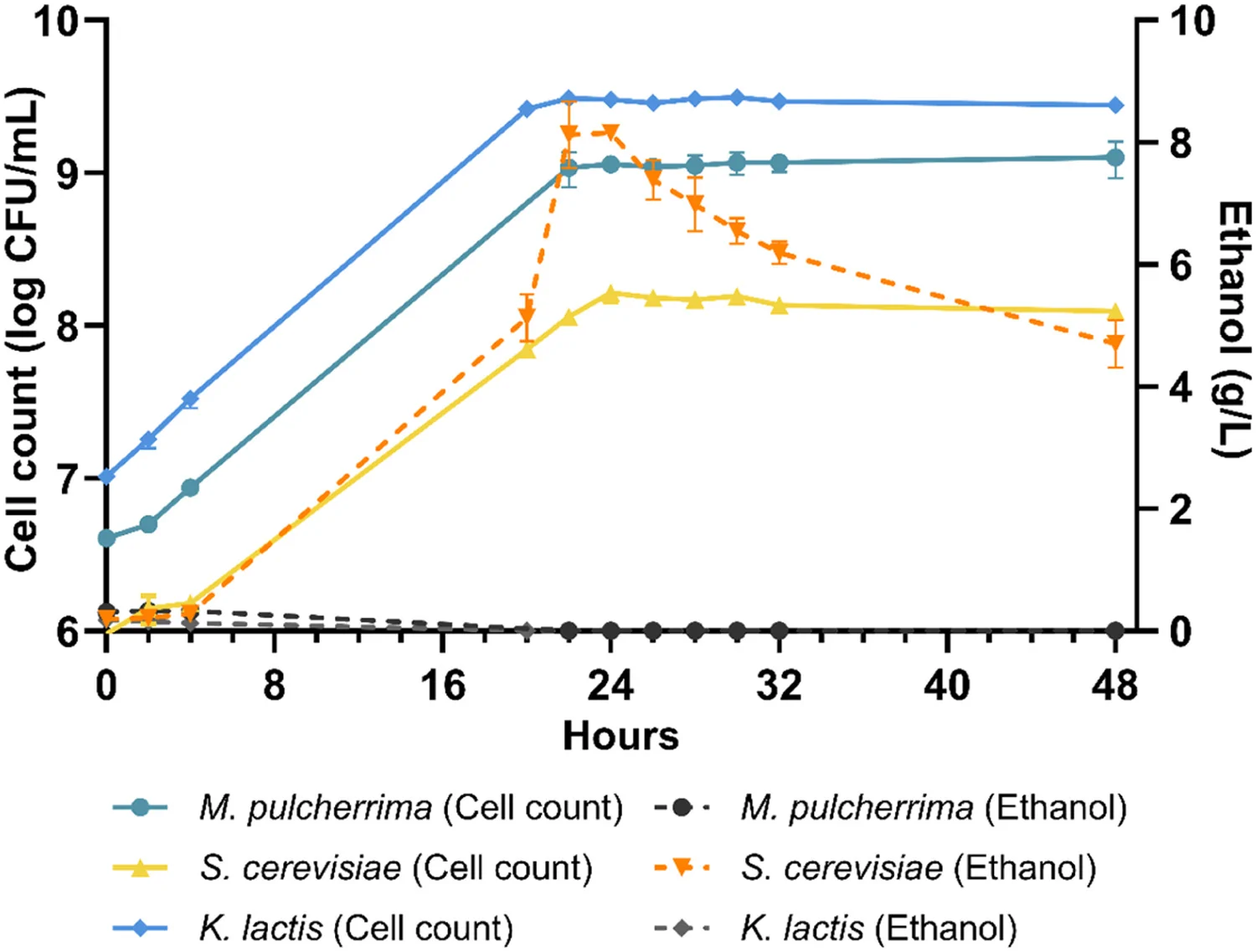
Cell growth (CFU/mL) and ethanol production of *Kluyveromyces lactis, Metschnikowia pulcherrima*, and *Saccharomyces cerevisiae* in medium containing 20 g/L glucose and 30 g/L yeast extract at initial of pH=5.0.

### 3.4 Optimization of pH and temperature using CCD

#### 3.4.1 Model fitting and statistical analysis

To optimize bioprocess conditions with respect to pH and temperature, a Box–Wilson Central Composite Design (CCD) was employed, which was first described by Box & Wilson [43]. Specifically, a Central Composite Circumscribed (CCC) design was selected, which integrates a full factorial design with center points and augments it with axial (star) points to capture curvature in the response surface. This design is particularly well-suited for modeling second-order effects due to its rotatability and spherical symmetry [40].

Using this approach, the effects of pH (range 3.59–6.41) and temperature (range 17.95 °C –32.05 °C) on viable cell count were evaluated, as detailed in Section 2.6. The resulting experimental data was fitted into a second-order polynomial model to describe the relationship between the response variable and the independent factors. The final regression equation, incorporating the estimated coefficients and coded variables, is **(Eq. 5)**:

$$\begin{array}{c} Y=1.35*10^{9}+0.1503*10^{9}X_{pH}-0.3644*10^{9}X_{\text{temp}\;}+0.1167*10^{9}X_{pH}X_{\text{temp}\;}\\ -0.0174*10^{9}{X_{pH}}^{2}-0.0646*10^{9}{X_{\text{temp}\;}}^{2} \end{array}$$

where **Y** is the predicted viable cell count (CFU/mL), while **X*ₚ_H_*** and **X*ₜₑₘₚ*** are coded values for pH and temperature. Table 5**b** shows the analysis of variance (ANOVA) of the response surface model, while Table 5**a** shows the summary of fit. The fitted quadratic model was statistically significant (**F(5,6) = 27.9287, p** **=** **0.0004**) with a high coefficient of determination (**R² = 0.959**), indicating good model fit and predictive ability.

**Table 5:** (a) Summary of Fit, (b) ANOVA of the RSM and (c) Effect Summary.

ANOVA identified temperature (**p** **<** **0.0001**) and pH (**p** **=** **0.0047**) as significant factors influencing VCC (Table 5c), while interaction between them was close to significance (**p** **=** **0.0535**). Quadratic terms for both factors were not significant (**p** **>** **0.14**), suggesting primarily linear effects within the tested range. Parameter estimates showed that increasing temperature within the range negatively impacted VCC, whereas pH was positively correlated. The interaction term suggested a synergistic effect, where the impact of pH varied slightly depending on temperature.

Optimization of the response surface predicted maximum cell density at approximately pH 4.59 and temperature 17.95 °C, 1.74×10^9^ CFU/mL which was validated experimentally.

#### 3.4.2 Response surface analysis

To explore how pH and temperature affect viable cell count, a 3D surface plot (Fig. 4) was generated from the fitted response surface model. The 3D surface showed that temperature had a strong influence on cell viability, with the strain performing better at lower temperatures, and showing a general decline at higher temperatures across all pH levels. At the lower end of the temperature range, pH had little effect, suggesting the strain is robust across a wide pH range. However, as temperature increased, the effect of pH became more noticeable, which is likely due to increased biological stress. Interestingly, the decline in VCC was less steep at higher pH values, suggesting a possible protective effect. The shape of the plot also suggested some curvature in the response, pointing to the presence of both linear and quadratic effects, even though not all terms were statistically significant.

**Fig. 4:**
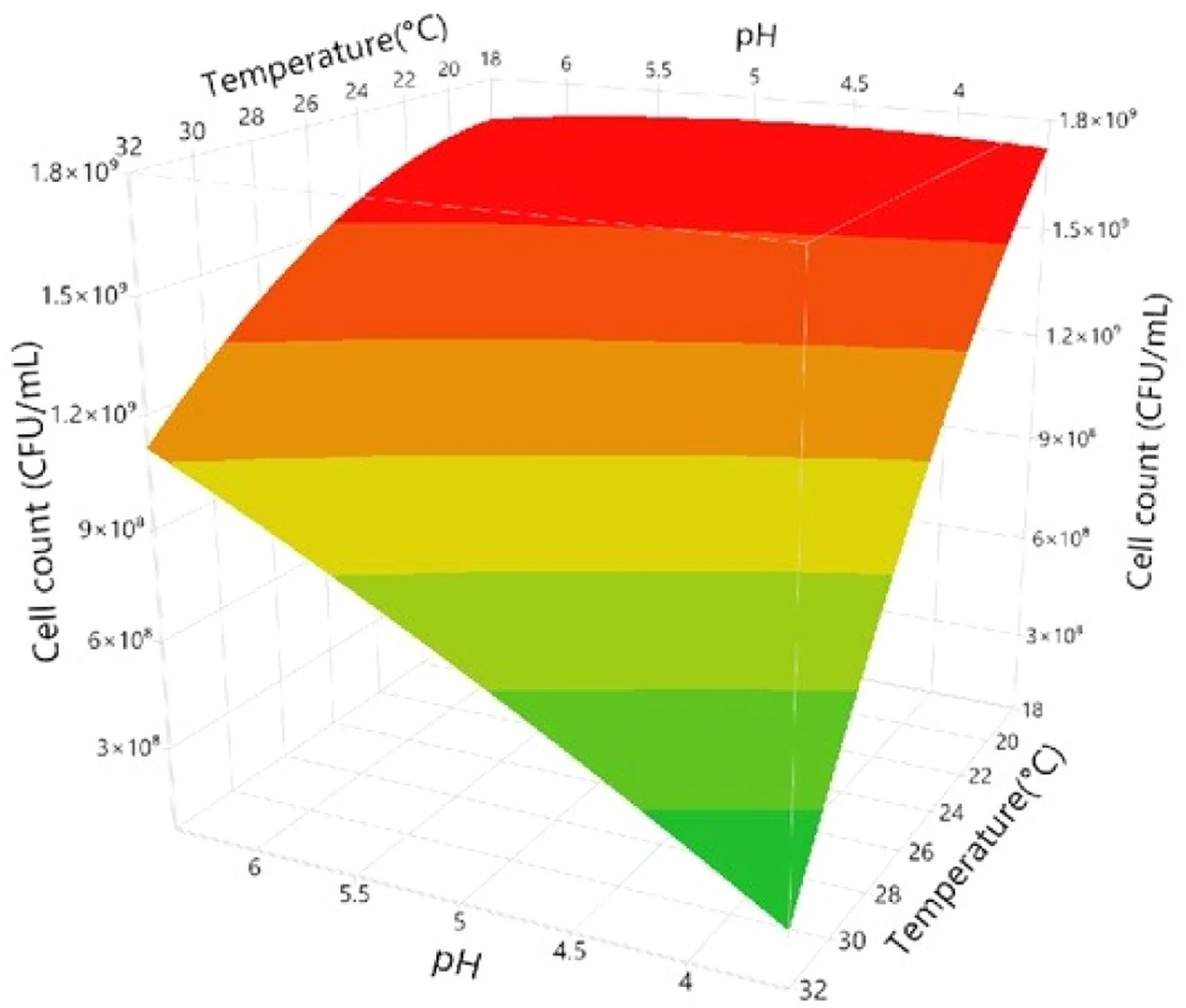
Response surface methodology (RSM) analysis illustrating the effects of pH and temperature on viable cell count: three-dimensional response surface plot generated from the fitted model.

#### 3.4.3 Model validation

To validate the model for maximizing the VCC, experiments were conducted in biological triplicates under the predicted optimal conditions (pH = 4.59, temperature = 17.95 °C). The model had a predicted value of 1.74 ×10^9^ CFU/mL (95% confidence interval: 1.53×10^9^ - 1.94×10^9^ CFU/mL). The observed responses closely matched the predicted value, with a VCC of 1.70 ± 0.05 ×10^9^ CFU/mL (mean ± SD, n = 3). A one-sample *t*-test comparing the experimental mean with the predicted value showed no significant difference **(t(2) = −1.193, p** **=** **0.355)**, supporting the model’s reliability.

Although the terms Xₚₕ², Xₜₑₘₚ², and the interaction term XₚₕXₜₑₘₚ were not individually significant at the 95% confidence level, they were retained in the final model. Retaining these terms improved R² (0.958 vs. 0.899) and adjusted R² (0.924 vs. 0.877) relative to the reduced model, while predicted residual error sum of squares (PRESS) remained comparable between the full and reduced models. The full quadratic model is generally preferred unless removing terms produces a substantial improvement in PRESS and R² ([44], p. 52); since no such improvement was observed here, the full model was retained. This is consistent with a systematic comparison of model-reduction strategies across published RSM studies, which found the full second-order model to be a reasonable default for analysis [45]. This decision was further supported by better agreement between predicted and experimentally validated responses. This suggests that these terms may capture subtle curvature effects near the optimum that are not fully reflected in the statistical tests. Therefore, they were retained in the final model to preserve hierarchical structure and enhance predictive performance.

The model was also tested in 10 L (6 L working volume) bioreactors in triplicates under another set of conditions within the modelled range (20 °C, pH=6). The observed responses closely matched the predicted values, 1.64 ± 0.06×10^9^ CFU/mL (mean ± SD, n = 3), compared to the model prediction of 1.67×10^9^ CFU/mL (95% confidence interval: 1.48×10^9^ - 1.85×10^9^ CFU/mL). A one-sample *t*-test comparing the experimental mean of the predicted value showed no significant difference **(t(2) = 0.6182, p** **=** **0.5995)**, indicating that the observed value closely aligned with the predicted value even at the larger scale.

## 4 Discussion

The present study aimed to optimize the production of *Metschnikowia pulcherrima* APC 1.2 through systematic evaluation of carbon sources, nutrient ratios, pH, and temperature to achieve maximal viable cell count (VCC). In a previous optimization study, Spadaro et al. [29] reported enhanced growth of *M. pulcherrima* when using polyols such as d-mannitol and a mixture of d-mannitol and l-sorbose to sucrose, which was the best-performing carbon source in our study. However, it should be noted that their experiments were conducted at an unadjusted pH of approximately 6.83, whereas our initial pH was set to 5.0, which may have significantly influenced the observed outcomes. When we tested the most promising conditions from both studies, namely 20 g/L sucrose and 7.5 g/L mannitol, under unadjusted pH conditions (pH ≈ 6.8), our results aligned more closely with those of Spadaro et al. [29] (Fig. 1). These findings suggest that the optimal pH for promoting growth and substrate utilization may depend on the specific carbon source used, and may show a possible difference in carbon source preferences at a strain level, given the known heterogeneity of *M. pulcherrima* strains [[46], [47], [48]].

The results revealed that *M. pulcherrima* exhibits different VCC depending on the carbon source used, with disaccharides such as sucrose supported higher VCC than monosaccharides such as glucose, including an equimolar glucose-fructose mixture simulating full hydrolysis of sucrose (Fig. 1). A similar trend was reported by Spadaro et al. [29], with sucrose and maltose generally supporting higher VCC than the monosaccharides, although only the difference between sucrose and fructose at 20 g/L was statistically significant**.** This is somewhat surprising, given the low invertase activity previously reported in *M. pulcherrima* [27,35].

This strain's genome was previously sequenced [19], and subsequently examined by Tatay-Núñez et al. [49] who found no *S. cerevisiae* ortholog of SUC2 but identified glucoamylases and α-glucosidases that may contribute to intracellular sucrose hydrolysis. Our own analysis of the same genomic sequence, benchmarked against the *S. cerevisiae* S288C reference genome [50], likewise confirmed the absence of a SUC2 ortholog (see Supplementary 2). However, the absence of SUC2 does not necessarily prevent sucrose hydrolysis. Analysis of alternative sucrose-hydrolysis genes from IMA and MAL families identified two candidate α-glucosidase homologs in APC 1.2, supported by reciprocal BLAST analysis). These findings suggest that alternative pathways for sucrose hydrolysis may be present in the strain itself. Deng et al. [51] demonstrated that IMA proteins exhibit broad substrate specificity and are capable of hydrolyzing sucrose in vitro. At high substrate concentrations, sucrose was the best-preforming substrate for Ima3p. Marques et al. [52] provided genetic evidence that sucrose hydrolysis can occur in the absence of SUC2. In that study, a strain lacking SUC2, MAL12, MAL22, and MAL32 was still able to grow on sucrose if some sucrose-transporter genes are present (MAL11, MAL21, MAL31, MPH2, MPH3), with increased sucrose hydrolysis activity accompanied by upregulation of IMA1 and IMA5. However, the deletion of all five IMA genes (IMA1–IMA5) resulted in a complete loss of sucrose hydrolysis and growth on sucrose, suggesting that the IMA family collectively constitutes an alternative metabolic pathway for sucrose hydrolysis. However, the presence of an alternative sucrose-hydrolysis pathway does not explain why sucrose resulted in a higher VCC than glucose. In fact, this pathway requires an additional energy investment, as proton symport of disaccharides costs roughly 1 ATP per molecule transported, compared to facilitated diffusion of monosaccharides, which is passive and energy-free [53,54].

*M. pulcherrima* also has strong orthologs for glucose transport (HXT-family) and sensing (SNF3/RGT2), suggesting that the observed difference is unlikely to be explained solely by the absence of glucose transport or sensing machinery. The core components of the glucose-repression pathway (including SNF1/SNF4 and MIG1) are also present. Another related possibility is that rapid glucose influx could outpace downstream metabolic capacity, resulting in greater fluctuations in glycolytic intermediate concentrations and potentially diverting carbon towards overflow products, whereas sucrose's gated intracellular release may avoid this [55,56]. Further research is required to verify this hypothesis.

Optimization of the sucrose-to-yeast extract ratio demonstrated a strong dependency on the amount of yeast extract within the tested range, with linear increase in VCC before reaching a plateau (Fig. 2; Supplementary 1). The contribution of carbon from yeast extract can be excluded as a primary driver of this linear trend, as the maximal increase in carbon content across the tested range is too small (≤1.8 g total carbohydrates/L, calculated from the manufacturer's specification sheet) to account for the observed differences, given that a VCC was similar across all three sucrose concentrations tested (20, 35, 50 g/L).

Interestingly, Schnierda et al. [27] found no significant difference in biomass at yeast extract concentrations of 100 and 500 mg/L YAN. However, as their measurements focused on total biomass rather than VCC, yeast extract may affect VCC differently from biomass accumulation measured by dry weight. The trend observed here is similar to that reported by Spadaro et al. [29], where growth was tested using only yeast extract and no additional carbon source. However, the plateauing and subsequent decrease observed here cannot be explained by the Crabtree effect as proposed by Spadaro et al. [29], as we saw that the *M. pulcherrima* strain APC 1.2 did not exhibit Crabtree metabolism under the tested conditions.

As this plateauing occurs across conditions with or without additional sugars and at varying concentrations of sugars, one potential explanation is a metabolic imbalance arising from excess peptides and amino acids supplied by yeast extract. High concentrations of assimilable nitrogen can trigger Nitrogen Catabolite Repression (NCR), a regulatory mechanism that downregulates use of alternative nitrogen pathways/sources, limiting the yeasts flexibility in nitrogen assimilation [[57], [58], [59]]. However, NCR activity was not directly measured, and this interpretation should be regarded as a plausible hypothesis rather than a confirmed mechanism. Alternative explanations cannot be excluded, including inhibitory effects from excess intracellular amino acid accumulation [60], or other components of yeast extract, such as minerals or nucleic acid derivatives, acting independently of, or in addition to, nitrogen-related effects. Further work directly assessing NCR-related gene expression, combined with dose-response testing of individual yeast extract components (e.g., specific amino acids, minerals, or nucleotide derivatives), would help clarify their relative and combined contributions to the observed plateau.

While previous research demonstrated that *M. pulcherrima* exhibits Crabtree-negative behavior using molasses-based media with complex sugar compositions [27,34,35], our study aimed to validate and refine this finding using a defined, single-carbon-source design that eliminates the confounding effects of enzymatic sugar hydrolysis and mixed carbon sources. Our results confirm and extend these earlier findings under more controlled conditions, using glucose as the sole carbon source to provide a clearer, more reproducible framework for characterizing the Crabtree-negative phenotype in this species. These results have practical implications for bioprocess design: batch and fed-batch cultivation should yield comparable results for *M. pulcherrima*, provided the concentration of sugars or other media components remain below the threshold that triggers overflow metabolism or another sugar-related inhibitory effect. This was demonstrated in a study by Abeln & Chuck [20] who found no significant difference in final biomass yield between batch and fed-batch production.

Environmental parameter modeling using Central Composite Design (CCD) identified both pH and temperature as factors influencing growth (see Results, Table 5c). The optimal temperature range for maximizing the viable cell count was found to be around 18 - 22 °C, with a wide pH tolerance at this temperature (Fig. 4) which is generally consistent with previous studies. Yuan et al. [61] also identified an appropriate growth temperature range for their strain between 15 and 25 °C. The growth rate (µ_max_) peaked at approximately 20 °C, however, while total growth (area under the curve, AUC) was highest at 25 °C. Schnierda et al. [27] reported better growth at 25–30 °C than at 15–20 °C, likely due to the shorter incubation period of 24 h of the culture. Abeln & Chuck [21] reported a similar optimal temperature range between 15 and 25 °C for biomass and lipid production, with growth being significantly slower at 15 °C until maximum values were reached, a delay effect that we also observed. At our lowest tested temperature (17.95 °C), it took 28 h to reach the stationary phase while at 20 °C and above stationary phase was reached it within 24 h. At 20 °C, Abeln & Chuck [21] also found no difference in growth between pH 4 and 5 in the presence of glucose, consistent with our own result at low temperatures. Similarly, Spadaro et al. [29] reported a wide pH tolerance for *M. pulcherrima* (growth possible from pH 3.0–10.0), with the strongest growth observed at an initial pH between 5.0 and 7.5, in line with our own data. The response surface accurately describes growth within the experimentally tested range of pH and temperature. However, as the model is valid only within the studied framework, extrapolation beyond these conditions may lead to unrealistic predictions and should be interpreted with caution.

CCD suggests that the strain’s sensitivity to pH may be temperature-dependent, and that precise control of both parameters becomes more important under higher temperatures. These results are consistent with previous studies on *S. cerevisiae*, which have shown that high temperatures increase the minimum pH required for growth [62] and enhance the inhibitory effect of acetic acid on growth [63,64], though whether the same mechanism applies here remains to be tested.

The model showed strong predictive power and was validated experimentally, at both 1 L and 10 L reactor scales, confirming its scalability and utility for process control. Central composite design has shown to be a good and efficient method to obtain reliable results while reducing experimental workload and optimizing process performance. Overall, the response surface analysis supported the statistical findings and provided a clearer picture of how the two variables influence the growth.

## 5 Conclusions

This study identified and optimized cultivation and process conditions, that when taken together, maximize the viable cell count (VCC) of *M. pulcherrima* strain APC 1.2. This was achieved by taking a three-step approach: optimizing carbon and nitrogen sources, understanding the cells’ metabolism, and by adjusting environmental factors (pH and temperature) while accounting for their interactive effects. Firstly, 20 g/L sucrose has been shown to be the best carbon source among those tested for maximizing the viable cell count, with disaccharides generally outperforming monosaccharides. The optimal concentration yeast extract was found to be 1600 mg YAN/L, corresponding to approximately 30 g/L of yeast extract, after which no further increase in viable cell counts was measured. This cessation in growth occurred even at higher concentrations of sucrose, indicating that factors beyond carbon availability may limit growth under these conditions. We confirmed that *M. pulcherrima* is Crabtree-negative by showing the absence of ethanol production when grown on glucose as the sole carbon source under aerobic conditions, thereby supporting and refining previous findings under more controlled conditions. This finding has practical relevance for bioprocess development of *M. pulcherrima*, as it indicates that aerobic cultivation can be performed without ethanol formation, suggesting that batch operation at elevated sugar concentrations may be feasible without the need for fed-batch strategies solely to suppress Crabtree metabolism, provided that other inhibitory effects (e.g., osmotic stress) are not limiting. By using the central composite design, we found that the highest VCC were observed around 18 - 22 °C, with the VCC being stable across a broad pH range. As temperature increased, the strains sensitivity to acidic pH became more pronounced. The model was validated both in 1 L and 10 L bioreactors. The combination of systematic medium screening, experimental evaluation of the metabolic phenotype under defined conditions, and design-of-experiment-based optimization of cultivation parameters efficiently identifies optimal conditions with a limited number of bioreactor experiments. This structured workflow can be adapted to other non-conventional yeasts with limited prior physiological or process characterization.

## Funding sources

This project is funded by the Swiss National Science Foundation (SNSF): grant number 211502 to Florian Freimoser, Ueli von Ah and Sarrah Perren. The research was carried out at Agroscope.

## CRediT authorship contribution statement

**Bogdan T. Dinić:** Writing – original draft, Visualization, Validation, Methodology, Investigation, Formal analysis, Conceptualization. **Nicholas A. Bokulich:** Writing – review & editing, Supervision, Conceptualization. **Florian M. Freimoser:** Writing – review & editing, Resources, Funding acquisition. **Ueli von Ah:** Writing – review & editing, Supervision, Methodology, Funding acquisition, Conceptualization.

## Declaration of competing interest

The authors declare that they have no known competing financial interests or personal relationships that could have appeared to influence the work reported in this paper.

## Data Availability

Data will be made available on request.
