# Supplementary material for: Bioprocess optimization of Metschnikowia pulcherrima: carbon source selection, nutrient ratio tuning, and environmental parameter modeling using response surface methodology
Source: Biotechnol Rep (Amst). 2026 Oct 10;52:e00984. doi: 10.1016/j.btre.2026.e00984 (PMC13718474; doi:10.1016/j.btre.2026.e00984)
Supplement: Supplementary file 1 [file mmc1.docx]

Contents

[Supplementary Material 1 Statistical analysis for Section 3.2: Optimization of Sugar to Yeast Extract Ratio in Shake Flasks (mixed full factorial)	2](#_Toc243424854)

[Supplementary Material 2 Genomic BLAST screening of sucrose-, glucose orthologs in *Metschnikowia pulcherrima* strain APC 1.2	4](#_Toc243424855)

[S1. Methods	4](#_Toc243424856)

[S1.1 Genomic data sources	4](#_Toc243424857)

[S1.2 Candidate gene identification, extraction, and BLAST analysis	4](#_Toc243424858)

[S1.3 Software versions and custom scripts	4](#_Toc243424859)

[S2. Rationale for Candidate Gene Selection	5](#_Toc243424860)

[S3. Results Tables	6](#_Toc243424861)

[Tables S3.1. Top BLAST hit per query gene, with reciprocal BLAST validation	6](#_Toc243424862)

[Tables S3.2. Secondary and tertiary BLAST hits per query gene (supplementary to Tables S1-S3)	9](#_Toc243424863)

[S4. References	13](#_Toc243424864)

Supplementary Material 1
Statistical analysis for Section 3.2: Optimization of Sugar to Yeast Extract Ratio in Shake Flasks (mixed full factorial)

Viable cell counts (CFU/mL) from individual flasks (n = 3 biological replicates per condition; 45 observations in total) were log₁₀-transformed and fitted with a second-order response-surface model (JMP). All statistics below are on the log₁₀ scale. Cultures were grown in 500 mL DURAN® baffled Erlenmeyer flasks (DWK Life Sciences, Germany) containing 100 mL of medium and incubated at 25 °C, initial pH 5.0, with agitation at 170 rpm for 48 h in an orbital shaker.

#### **Table S1. Summary of Fit**

| **Statistic** | **Value** |
| --- | --- |
| R² | 0.886 |
| Adjusted R² | 0.872 |
| Root mean square error | 0.142 |
| Mean of response | 8.979 |
| Observations | 45 |

#### **Table S2. Analysis of Variance (ANOVA) for the Quadratic Regression Model**

| **Source** | **DF** | **Sum of Squares** | **Mean Square** | **F Ratio** | **Prob > F** |
| --- | --- | --- | --- | --- | --- |
| Model | 5 | 6.1332 | 1.2266 | 60.73 | <.0001* |
| Error | 39 | 0.7877 | 0.0202 |  |  |
| Lack Of Fit | 9 | 0.4986 | 0.0554 | 5.75 | 0.0001* |
| Pure Error | 30 | 0.2892 | 0.0096 |  |  |
| Total | 39 | 0.7877 |  |  |  |

**R^2^=0.886, Adjusted R^2^=0.872**

#### **Table S3. Effect Summary**

| **Source** | **Logworth** |  | **PValue** |
| --- | --- | --- | --- |
| Yeast Extract(100,2100) | 16.449 |  | 0.00000 |
| Yeast Extract*Yeast Extract | 11.326 |  | 0.00000 |
| Sucrose(20,50) | 0.326 |  | 0.47184 |
| Sucrose*Sucrose | 0.254 |  | 0.55745 |
| Sucrose*Yeast Extract | 0.224 |  | 0.59765 |

**Figure S1. Actual by predicted cell count**

Figure S1. Actual versus predicted log₁₀ cell count for all observations. Red line is the 1:1 reference.

**Figure S2. Residual by predicted**

Figure S2. Residuals versus predicted values; no funnel pattern remains after log₁₀ transformation.

**Figure S3. Response surface**

Figure S3. Response surface methodology (RSM) analysis of the effects of sucrose and yeast extract on viable cell count. Three-dimensional response surface generated from the fitted second-order model.

# Supplementary Material 2 Genomic BLAST screening of sucrose-, glucose orthologs in *Metschnikowia pulcherrima* strain APC 1.2

## S1. Methods

### S1.1 Genomic data sources

Predicted proteomes were obtained for *Metschnikowia aff. pulcherrima* strain APC 1.2 (BioProject [PRJNA508581](https://www.ncbi.nlm.nih.gov/bioproject/508581); genome assembly [GCA_004217705.1](https://www.ncbi.nlm.nih.gov/datasets/genome/GCA_004217705.1/), ASM421770v1) and *Saccharomyces cerevisiae* S288C (RefSeq proteome, genome assembly [GCF_000146045.2](https://www.ncbi.nlm.nih.gov/datasets/genome/GCF_000146045.2/), assembly name R64; BioProject [PRJNA128](https://www.ncbi.nlm.nih.gov/bioproject/128)).

### S1.2 Candidate gene identification, extraction, and BLAST analysis

Candidate *S. cerevisiae* genes of interest were compiled from the primary literature (see Section S2, 'Rationale for Gene Selection'). The official gene symbol, full protein name, RefSeq protein accession number (NP_), NCBI Gene ID, corresponding NCBI record link and general protein information were manually retrieved for each candidate gene from the NCBI Gene database. This was necessary because several genes were only generically annotated in the *S. cerevisiae* source proteome FASTA header (e.g. 'alpha-glucoside permease', 'glucose sensor', 'hexokinase'), precluding reliable identification by keyword search alone. This information was compiled into a reference table. The protein sequences corresponding to each verified NP accession were then extracted directly from the *S. cerevisiae* RefSeq proteome using the SeqIO module of Biopython and written to a combined query FASTA file.

A local protein BLAST database was constructed from the M. pulcherrima APC 1.2 predicted proteome using NCBI BLAST+ v2.17.0 (makeblastdb -dbtype prot) and the combined query file was searched against this database using blastp (-evalue 1e-5 -outfmt 6 -max_target_seqs 3, including the query and subject length fields). Query and subject percent coverage were calculated from the raw alignment coordinates as follows: Query coverage (%) = 100 × (qend − qstart + 1)/qlen; subject coverage (%) = 100 × (send − sstart + 1)/slen.

To validate orthology, the *M. pulcherrima* proteins returned by the forward search were extracted using Biopython and searched against a second local database built from the *S. cerevisiae* RefSeq proteome using blastp (-evalue 1e-5, -outfmt 6 and -max_target_seqs 1).

### S1.3 Software versions and custom scripts

- NCBI BLAST+ v2.17.0 (Windows)
- Biopython (SeqIO module) for sequence extraction
- Python 3

Custom Python scripts were used to extract query sequences by verified accession, identify and extract reciprocal-search target sequences from BLAST output, calculate alignment coverage, and construct the results tables described above. These tables were then adjusted manually for Tables S3.1.1–S3.2.3. These scripts are not independently published or peer-reviewed bioinformatics tools; they perform routine, non-algorithmic data handling steps using the Biopython library. The scripts, the raw BLAST output and the constructed results tables are provided in the accompanying Supplementary Data archive (Supplementary2_Data_MP_Dinic_2026.zip) deposited on Zenodo under DOI 10.5281/zenodo.22698070 and are organised as follows: 01_Scripts (custom Python scripts); 02_Raw_Results (raw BLAST and reciprocal BLAST output and constructed results tables); and 03_Supplementary_Documents (this document and related supplementary files).

## S2. Rationale for Candidate Gene Selection

The candidate genes were selected based on their established roles in sucrose and disaccharide metabolism, hexose transport and glucose repression signalling in *S. cerevisiae*, as summarised below.

**Sucrose/maltose/isomaltose pathway gene candidates**

The genes SUC2, IMA1–IMA5, MAL12, MAL32, MAL11 (AGT1), MAL31, MPH2 and MPH3 were chosen based on their established roles in sucrose and alpha-glucoside metabolism. SUC2 was initially investigated as the primary sucrose-hydrolysis gene. Marques et al. (2017) revealed that IMA1/IMA5 upregulation sustains sucrose utilisation in SUC2/MAL-deficient strains, and that combined deletion of all five IMA genes eliminates this ability. The same study also showed that deleting MAL12, MAL22 and MAL32 alongside SUC2 was necessary to eliminate residual sucrose hydrolysis. This implicates this gene family in the SUC2-independent route. MPH2 and MPH3 (YDL247w and YJR160c) were added based on the work of Day et al. (2002), who characterised them as alpha-glucoside transporters via heterologous complementation.

**Glucose transport and sensing**

All 20 members of the HXT hexose transporter family (HXT1–17, GAL2, and the sensors SNF3/RGT2) (Özcan & Johnston, 1999) were tested to assess whether limited glucose transport capacity could explain the strain's preference for sucrose over glucose. Although Diderich et al. (1999) found that HXT1–7 exhibited the strongest activity and were the only members whose transcription correlated directly with extracellular glucose concentration (with GAL2, SNF3, RGT2 and HXT8–17 being expressed only weakly or under specific conditions), Wieczorke et al. (1999) showed that deleting only HXT1–7 was insufficient to block the uptake of glucose, mannose and fructose. In their CEN.PK2-1C-derived strain, complete abolition of hexose transport required the deletion of all HXT1–17, as well as MAL11, MPH2 and MPH3 (also known as AGT1, YDL247w and YJR160c, respectively). This confirms that the remaining transporters, which are individually minor, are collectively sufficient to sustain uptake. Therefore, all 20 family members were tested here rather than restricting the analysis to HXT1–7. HXT8–17 have received less attention, and many of their assigned functions are not directly related to sugar utilisation. However, expression of all except HXT11 and HXT12 is glucose-regulated (Rintala et al., 2008) ), and some, including HXT13, HXT15, HXT16 and HXT17, have been specifically linked to hexitol transport with distinct substrate specificities (Jordan et al., 2016). HXT12 and HXT14 were excluded from testing as they are absent/non-functional in the S288C reference strain.

**Core glucose-repression signaling pathway**

The candidate genes SNF1, SNF4, MIG1, HXK1, HXK2, GLK1, REG1, GLC7, TUP1, SNF3, and RGT2 were selected based on their established roles in the glucose repression pathway of *S. cerevisiae* based on reviews of Busti et al. (2010), and Kayikci & Nielsen, (2015). The core catalytic complex, which is formed by SNF1 and its regulatory subunit SNF4, is activated upon glucose limitation to phosphorylate and inactivate MIG1, relieving the repression of glucose-repressed genes. MIG1 mediates this repression in conjunction with the general co-repressor TUP1 (via its obligatory partner SSN6/CYC8). Through its nuclear association with MIG1 at target promoters, such as SUC2 GLK1 and HXK1 were kept as hexokinase-family homologues to act as parallel comparison points, despite not being directly involved in this signalling function. SNF1 is rendered inactive by the phosphatase complex formed by REG1 and GLC7, which serves as the pathway's 'off-switch'. SNF3 and RGT2 are upstream regulators and are included in the above group (glucose transport and sensing (HXT family, GAL2, SNF3/RGT2-type sensors)). Additional pathway components identified in both reviews include RGT1, MTH1, STD1, SAK1, ELM1, TOS3, MIG2, ADR1 and CAT8. These components were not included in the present analysis, but represent candidates for a broader analysis in the future.

## S3. Results Tables

### Tables S3.1. Top BLAST hit per query gene, with reciprocal BLAST validation

For each S. cerevisiae query gene, the single top-scoring hit against the *M. pulcherrima* proteome is reported, with percent identity, query- and subject-side coverage, and bit score. Each M. pulcherrima hit was also searched back against the *S. cerevisiae* proteome (reciprocal BLAST); the resulting top reciprocal hit and its percent identity are shown in the final two columns, alongside a verdict on orthology confirmation

#### Table S3.1.1. Sucrose/maltose/isomaltose pathway gene candidates

| **Gene** | **SC accession** | **MP top hit** | **MP annotation** | **Identity (%)** | **Query cov (%)** | **Subject cov (%)** | **Bit score** | **Reciprocal SC hit** | **Reciprocal identity (%)** |
| --- | --- | --- | --- | --- | --- | --- | --- | --- | --- |
| SUC2 | NP_012104.1 | NO HIT | n/a | - | - | - | - | n/a | - |
| IMA1 | NP_011803.3 | QBM87903.1 | alpha-glucosidase | 47.5 | 97.5 | 94.4 | 546 | IMA1 | 47.5 |
| IMA2 | NP_014485.1 | QBM87903.1 | alpha-glucosidase | 47.8 | 97.5 | 94.4 | 558 | IMA1 | 47.5 |
| IMA3 | NP_012096.1 | QBM87903.1 | alpha-glucosidase | 47.7 | 97.5 | 94.4 | 553 | IMA1 | 47.5 |
| IMA4 | NP_012314.1 | QBM87903.1 | alpha-glucosidase | 47.7 | 97.5 | 94.4 | 553 | IMA1 | 47.5 |
| IMA5 | NP_012319.1 | QBM87903.1 | alpha-glucosidase | 45.2 | 98.5 | 94.6 | 528 | IMA1 | 47.5 |
| MAL12 | NP_011808.3 | QBM87903.1 | alpha-glucosidase | 47.6 | 97.8 | 94.6 | 532 | IMA1 | 47.5 |
| MAL32 | NP_009858.3 | QBM87903.1 | alpha-glucosidase | 47.7 | 97.8 | 94.6 | 533 | IMA1 | 47.5 |
| MAL11  (= AGT1) | NP_011805.3 | QBM90166.1 | MFS transporter, SP family, general alpha glucoside:H+ symporter | 44.6 | 89.1 | 95 | 503 | MPH3 | 46.0 |
| MAL31 | NP_009857.1 | QBM90166.1 | MFS transporter, SP family, general alpha glucoside:H+ symporter | 43.8 | 91.5 | 97.7 | 514 | MPH3 | 46.0 |
| MPH2 | NP_010034.1 | QBM90166.1 | MFS transporter, SP family, general alpha glucoside:H+ symporter | 47.3 | 88 | 92.9 | 518 | MPH3 | 46.0 |
| MPH3 | NP_012694.1 | QBM90166.1 | MFS transporter, SP family, general alpha glucoside:H+ symporter | 46 | 92.7 | 96.7 | 520 | MPH3 | 46.0 |

#### Table S3.1.2. Glucose transport and sensing

| **Gene** | **SC accession** | **MP top hit** | **MP annotation** | **Identity (%)** | **Query cov (%)** | **Subject cov (%)** | **Bit score** | **Reciprocal SC hit** | **Reciprocal identity (%)** |
| --- | --- | --- | --- | --- | --- | --- | --- | --- | --- |
| HXT1 | NP_011962.1 | QBM86508.1 | MFS transporter, SP family, sugar:H+ symporter | 57.4 | 93.3 | 98.7 | 639 | HXT6 | 58.9 |
| HXT2 | NP_013724.1 | QBM87556.1 | MFS transporter, SP family, sugar:H+ symporter | 62.4 | 91.9 | 91.2 | 643 | HXT6 | 60.5 |
| HXT3 | NP_010632.1 | QBM86508.1 | MFS transporter, SP family, sugar:H+ symporter | 57.7 | 92.6 | 96.9 | 639 | HXT6 | 58.9 |
| HXT4 | NP_011960.2 | QBM86508.1 | MFS transporter, SP family, sugar:H+ symporter | 58.9 | 94.8 | 99.8 | 656 | HXT6 | 58.9 |
| HXT5 | NP_011964.1 | QBM86508.1 | MFS transporter, SP family, sugar:H+ symporter | 60.1 | 88.5 | 96.9 | 657 | HXT6 | 58.9 |
| HXT6 | NP_010630.1 | QBM86508.1 | MFS transporter, SP family, sugar:H+ symporter | 58.9 | 95.8 | 99.1 | 673 | HXT6 | 58.9 |
| HXT7 | NP_010629.3 | QBM86508.1 | MFS transporter, SP family, sugar:H+ symporter | 58.7 | 95.8 | 99.1 | 671 | HXT6 | 58.9 |
| HXT8 | NP_012321.1 | QBM87556.1 | MFS transporter, SP family, sugar:H+ symporter | 59.3 | 90.5 | 94.2 | 627 | HXT6 | 60.5 |
| HXT9 | NP_012316.1 | QBM86508.1 | MFS transporter, SP family, sugar:H+ symporter | 58.4 | 95.6 | 98.9 | 655 | HXT6 | 58.9 |
| HXT10 | NP_116644.1 | QBM87556.1 | MFS transporter, SP family, sugar:H+ symporter | 57.4 | 97.1 | 96.4 | 616 | HXT6 | 60.5 |
| HXT11 | NP_014486.1 | QBM86508.1 | MFS transporter, SP family, sugar:H+ symporter | 59.3 | 93.8 | 97.1 | 651 | HXT6 | 58.9 |
| HXT13 | NP_010845.1 | QBM86508.1 | MFS transporter, SP family, sugar:H+ symporter | 49.4 | 94.9 | 98.7 | 554 | HXT6 | 58.9 |
| HXT15 | NP_010036.1 | QBM86508.1 | MFS transporter, SP family, sugar:H+ symporter | 51.1 | 92.2 | 96.5 | 553 | HXT6 | 58.9 |
| HXT16 | NP_012692.3 | QBM86508.1 | MFS transporter, SP family, sugar:H+ symporter | 50.9 | 92.2 | 96.5 | 550 | HXT6 | 58.9 |
| HXT17 | NP_014470.1 | QBM86508.1 | MFS transporter, SP family, sugar:H+ symporter | 49.3 | 94.9 | 98.7 | 551 | HXT6 | 58.9 |
| GAL2 | NP_013182.1 | QBM86508.1 | MFS transporter, SP family, sugar:H+ symporter | 56.6 | 94.6 | 99.1 | 632 | HXT6 | 58.9 |
| SNF3 | NP_010087.1 | QBM86342.1 | MFS transporter, SP family, sugar:H+ symporter | 55.7 | 56.1 | 67 | 583 | SNF3 | 55.7 |
| RGT2 | NP_010143.1 | QBM86342.1 | MFS transporter, SP family, sugar:H+ symporter | 56.6 | 62.5 | 65.1 | 573 | SNF3 | 55.7 |

#### Table S3.1.3. Core glucose-repression signaling pathway

| **Gene** | **SC accession** | **MP top hit** | **MP annotation** | **Identity (%)** | **Query cov (%)** | **Subject cov (%)** | **Bit score** | **Reciprocal SC hit** | **Reciprocal identity (%)** |
| --- | --- | --- | --- | --- | --- | --- | --- | --- | --- |
| SNF1 | NP_010765.3 | QBM87938.1 | carbon catabolite-derepressing protein kinase | 64.6 | 95.1 | 94.9 | 776 | SNF1 | 66.0 |
| SNF4 | NP_011400.1 | QBM89960.1 | CBS domain-containing protein | 66.6 | 98.1 | 93.8 | 441 | SNF4 | 66.6 |
| MIG1 | NP_011480.1 | QBM89872.1 | zinc-finger protein CreA/MIG | 55.7 | 18.5 | 20.8 | 122 | MIG1 | 55.7 |
| HXK1 | NP_116711.3 | QBM90772.1 | hexokinase | 68.9 | 99.4 | 99.4 | 706 | HXK1 | 67.5 |
| HXK2 | NP_011261.1 | QBM90772.1 | hexokinase | 68.2 | 99.4 | 99.4 | 700 | HXK1 | 67.5 |
| GLK1 | NP_009890.1 | QBM88337.1 | hexokinase | 48.6 | 97 | 97.7 | 445 | GLK1 | 48.6 |
| REG1 | NP_010311.1 | QBM86900.1 | protein of unknown function DUF1752 | 36.4 | 33.9 | 40.7 | 194 | REG1 | 36.4 |
| GLC7 | NP_011059.3 | QBM90616.1 | serine/threonine-protein phosphatase PP1 catalytic subunit | 91.8 | 97.4 | 92.7 | 583 | GLC7 | 91.8 |
| TUP1 | NP_010007.1 | QBM90872.1 | glucose repression regulatory protein TUP1 | 53.6 | 73.8 | 75.7 | 516 | TUP1 | 58.2 |
| CYC8 | NP_009670.3 | QBM87506.1 | glucose repression mediator protein | 68 | 38.3 | 39.8 | 567 | CYC8 | 61.1 |

Tables S3.2. Secondary and tertiary BLAST hits per query gene (supplementary to Tables S1-S3)
This table lists the second- and third-ranked BLAST hits for each query gene, if available, as well as the top hit already reported in Tables S3.1. Only genes with more than one significant hit are shown beyond the first columns. Cells filled with n/a indicate that fewer than three significant hits were returned for that gene. As with Table S3.1, the results are presented in three subtables

#### Table S3.2.1. Sucrose/maltose/isomaltose pathway secondary and tertiary BLAST hits

| **Gene** | **MP hit 2** | **MP annotation 2** | **Identity 2 (%)** | **Query cov 2 (%)** | **Subject cov 2 (%)** | **Bit score 2** | **MP hit 3** | **MP annotation 3** | **Identity 3 (%)** | **Query cov 3 (%)** | **Subject cov 3 (%)** | **Bit score 3** |
| --- | --- | --- | --- | --- | --- | --- | --- | --- | --- | --- | --- | --- |
| SUC2 | n/a | n/a | n/a | n/a | n/a | n/a | n/a | n/a | n/a | n/a | n/a | n/a |
| IMA1 | QBM90856.1 | Alpha amylase, catalytic domain | 41 | 54.8 | 98.1 | 246 | n/a | n/a | n/a | n/a | n/a | n/a |
| IMA2 | QBM90856.1 | Alpha amylase, catalytic domain | 41.4 | 54.8 | 98.1 | 243 | n/a | n/a | n/a | n/a | n/a | n/a |
| IMA3 | QBM90856.1 | Alpha amylase, catalytic domain | 41.4 | 54.8 | 98.1 | 243 | n/a | n/a | n/a | n/a | n/a | n/a |
| IMA4 | QBM90856.1 | Alpha amylase, catalytic domain | 41.4 | 54.8 | 98.1 | 243 | n/a | n/a | n/a | n/a | n/a | n/a |
| IMA5 | QBM90856.1 | Alpha amylase, catalytic domain | 39.3 | 55.9 | 99.7 | 227 | QBM88055.1 | 1,4-alpha-glucan branching enzyme | 24.2 | 31.7 | 21.5 | 47 |
| MAL12 | QBM90856.1 | Alpha amylase, catalytic domain | 41 | 55.5 | 99.7 | 233 | n/a | n/a | n/a | n/a | n/a | n/a |
| MAL32 | QBM90856.1 | Alpha amylase, catalytic domain | 41.3 | 55.5 | 99.7 | 234 | n/a | n/a | n/a | n/a | n/a | n/a |
| MAL11 (= AGT1) | QBM85577.1 | MFS transporter, sugar porter (SP) family | 25.5 | 76.1 | 88.7 | 110 | QBM88660.1 | MFS transporter, sugar porter (SP) family | 26.6 | 66.9 | 77.7 | 101 |
| MAL31 | QBM86508.1 | MFS transporter, SP family, sugar:H+ symporter | 23.7 | 82.4 | 93.4 | 99 | QBM85602.1 | MFS transporter, sugar porter (SP) family | 24.2 | 74.3 | 85 | 99 |
| MPH2 | QBM86508.1 | MFS transporter, SP family, sugar:H+ symporter | 25.8 | 68.6 | 78.9 | 98 | QBM85602.1 | MFS transporter, sugar porter (SP) family | 24.1 | 75.5 | 85.6 | 97 |

#### Table S3.2.2. Glucose transport and sensing secondary and tertiary BLAST hits

| **Gene** | **MP hit 2** | **MP annotation 2** | **Identity 2 (%)** | **Query cov 2 (%)** | **Subject cov 2 (%)** | **Bit score 2** | **MP hit 3** | **MP annotation 3** | **Identity 3 (%)** | **Query cov 3 (%)** | **Subject cov 3 (%)** | **Bit score 3** |
| --- | --- | --- | --- | --- | --- | --- | --- | --- | --- | --- | --- | --- |
| HXT1 | QBM87556.1 | MFS transporter, SP family, sugar:H+ symporter | 56.1 | 94.7 | 97.1 | 624 | QBM85602.1 | MFS transporter, sugar porter (SP) family | 37.9 | 87.4 | 91.6 | 321 |
| HXT2 | QBM86508.1 | MFS transporter, SP family, sugar:H+ symporter | 60.5 | 95.7 | 95.8 | 641 | QBM85602.1 | MFS transporter, sugar porter (SP) family | 37 | 88.4 | 89 | 319 |
| HXT3 | QBM87556.1 | MFS transporter, SP family, sugar:H+ symporter | 59.8 | 88.9 | 92 | 635 | QBM85602.1 | MFS transporter, sugar porter (SP) family | 38.6 | 86.4 | 91.4 | 328 |
| HXT4 | QBM87556.1 | MFS transporter, SP family, sugar:H+ symporter | 60.3 | 89.6 | 92.9 | 642 | QBM85602.1 | MFS transporter, sugar porter (SP) family | 37 | 89.8 | 96.6 | 324 |
| HXT5 | QBM87556.1 | MFS transporter, SP family, sugar:H+ symporter | 59.1 | 84.6 | 92 | 634 | QBM85602.1 | MFS transporter, sugar porter (SP) family | 36.2 | 85.1 | 93.9 | 317 |
| HXT6 | QBM87556.1 | MFS transporter, SP family, sugar:H+ symporter | 60.5 | 92.3 | 95.3 | 669 | QBM85602.1 | MFS transporter, sugar porter (SP) family | 38.2 | 86.1 | 91.4 | 312 |
| HXT7 | QBM87556.1 | MFS transporter, SP family, sugar:H+ symporter | 60.3 | 92.3 | 95.3 | 667 | QBM85602.1 | MFS transporter, sugar porter (SP) family | 37.8 | 87.9 | 93.5 | 311 |
| HXT8 | QBM86508.1 | MFS transporter, SP family, sugar:H+ symporter | 58.5 | 90.7 | 95.2 | 616 | QBM85602.1 | MFS transporter, sugar porter (SP) family | 35.8 | 91.2 | 95.8 | 302 |
| HXT9 | QBM87556.1 | MFS transporter, SP family, sugar:H+ symporter | 59.3 | 94.2 | 95.8 | 645 | QBM85602.1 | MFS transporter, sugar porter (SP) family | 36.5 | 85.9 | 90.9 | 296 |
| HXT10 | QBM86508.1 | MFS transporter, SP family, sugar:H+ symporter | 59.5 | 89.9 | 90.8 | 607 | QBM85602.1 | MFS transporter, sugar porter (SP) family | 37.2 | 86.6 | 88 | 314 |
| HXT11 | QBM87556.1 | MFS transporter, SP family, sugar:H+ symporter | 58 | 97.5 | 99.8 | 649 | QBM85602.1 | MFS transporter, sugar porter (SP) family | 36.5 | 86.2 | 91.3 | 297 |
| HXT13 | QBM87556.1 | MFS transporter, SP family, sugar:H+ symporter | 52.6 | 88.3 | 92.2 | 551 | QBM85602.1 | MFS transporter, sugar porter (SP) family | 35.8 | 86.5 | 90.7 | 290 |
| HXT15 | QBM87556.1 | MFS transporter, SP family, sugar:H+ symporter | 50.4 | 95.4 | 99.8 | 552 | QBM85602.1 | MFS transporter, sugar porter (SP) family | 37.1 | 83.1 | 87.5 | 288 |
| HXT16 | QBM87556.1 | MFS transporter, SP family, sugar:H+ symporter | 50.2 | 95.4 | 99.8 | 548 | QBM85602.1 | MFS transporter, sugar porter (SP) family | 37.1 | 83.1 | 87.5 | 286 |
| HXT17 | QBM87556.1 | MFS transporter, SP family, sugar:H+ symporter | 53.7 | 86 | 88.9 | 548 | QBM85602.1 | MFS transporter, sugar porter (SP) family | 36.2 | 86.5 | 89.4 | 289 |
| GAL2 | QBM87556.1 | MFS transporter, SP family, sugar:H+ symporter | 60.5 | 87.3 | 90.7 | 630 | QBM85602.1 | MFS transporter, sugar porter (SP) family | 37.9 | 84.8 | 90.9 | 305 |
| SNF3 | QBM85602.1 | MFS transporter, sugar porter (SP) family | 48.6 | 54.4 | 90.9 | 474 | QBM86508.1 | MFS transporter, SP family, sugar:H+ symporter | 32.4 | 52.6 | 87.7 | 252 |
| RGT2 | QBM85602.1 | MFS transporter, sugar porter (SP) family | 48 | 61.7 | 88.2 | 475 | QBM86508.1 | MFS transporter, SP family, sugar:H+ symporter | 33.3 | 61.3 | 87.1 | 272 |

#### Table S3.2.3. Core glucose-repression signaling pathway secondary and tertiary BLAST hits

| **Gene** | **MP hit 2** | **MP annotation 2** | **Identity 2 (%)** | **Query cov 2 (%)** | **Subject cov 2 (%)** | **Bit score 2** | **MP hit 3** | **MP annotation 3** | **Identity 3 (%)** | **Query cov 3 (%)** | **Subject cov 3 (%)** | **Bit score 3** |
| --- | --- | --- | --- | --- | --- | --- | --- | --- | --- | --- | --- | --- |
| SNF1 | QBM88370.1 | serine/threonine-protein kinase GIN4 | 46.2 | 43.3 | 21.8 | 265 | QBM88815.1 | serine/threonine-protein kinase HSL1, negative regulator of Swe1 kinase | 45.3 | 42 | 19.6 | 250 |
| SNF4 | n/a | n/a | n/a | n/a | n/a | n/a | n/a | n/a | n/a | n/a | n/a | n/a |
| MIG1 | QBM86280.1 | zinc-finger protein CreA/MIG | 64.9 | 14.7 | 12.7 | 113 | QBM89661.1 | hypothetical protein METSCH_D07410 | 42.4 | 19.6 | 46.9 | 81 |
| HXK1 | QBM88337.1 | hexokinase | 39.2 | 93.4 | 99.2 | 322 | QBM87854.1 | hexokinase | 24 | 79 | 77.5 | 92 |
| HXK2 | QBM88337.1 | hexokinase | 40.4 | 91.6 | 97.3 | 320 | QBM87854.1 | hexokinase | 28.2 | 64.6 | 62.1 | 105 |
| GLK1 | QBM90772.1 | hexokinase | 37.2 | 97.2 | 92.1 | 264 | QBM87854.1 | hexokinase | 25.5 | 89 | 83.1 | 114 |
| REG1 | QBM90416.1 | hypothetical protein METSCH_E06650 | 53.8 | 3.8 | 10.8 | 53 | n/a | n/a | n/a | n/a | n/a | n/a |
| GLC7 | QBM87224.1 | serine/threonine-protein phosphatase PP1 catalytic subunit | 61.4 | 99.4 | 62.8 | 389 | QBM89050.1 | serine/threonine-protein phosphatase PP1 catalytic subunit | 59.5 | 93.3 | 55.6 | 388 |
| TUP1 | QBM90775.1 | transcription initiation factor TFIID subunit 5 | 31 | 26.1 | 21.9 | 110 | QBM90775.1 | transcription initiation factor TFIID subunit 5 | 24.9 | 38.8 | 34.6 | 102 |
| CYC8 | QBM87276.1 | Tetratricopeptide (TPR) repeat | 29.7 | 17.3 | 26.1 | 84 | QBM90366.1 | anaphase-promoting complex subunit 8 | 27.6 | 16.1 | 26.6 | 51 |

## S4. References

Busti, S., Coccetti, P., Alberghina, L., & Vanoni, M. (2010). Glucose Signaling-Mediated Coordination of Cell Growth and Cell Cycle in Saccharomyces Cerevisiae. *Sensors*, *10*(6), 6195–6240. https://doi.org/10.3390/s100606195

Day, R. E., Higgins, V. J., Rogers, P. J., & Dawes, I. W. (2002). Characterization of the putative maltose transporters encoded by YDL247w and YJR160c. *Yeast*, *19*(12), 1015–1027. https://doi.org/10.1002/yea.894

Diderich, J. A., Schepper, M., Van Hoek, P., Luttik, M. A. H., Van Dijken, J. P., Pronk, J. T., Klaassen, P., Boelens, H. F. M., De Mattos, M. J. T., Van Dam, K., & Kruckeberg, A. L. (1999). Glucose Uptake Kinetics and Transcription of HXTGenes in Chemostat Cultures of Saccharomyces cerevisiae. *Journal of Biological Chemistry*, *274*(22), 15350–15359. https://doi.org/10.1074/jbc.274.22.15350

Jordan, P., Choe, J.-Y., Boles, E., & Oreb, M. (2016). Hxt13, Hxt15, Hxt16 and Hxt17 from Saccharomyces cerevisiae represent a novel type of polyol transporters. *Scientific Reports*, *6*(1), 23502. https://doi.org/10.1038/srep23502

Kayikci, Ö., & Nielsen, J. (2015). Glucose repression in Saccharomyces cerevisiae. *FEMS Yeast Research*, *15*(6), fov068. https://doi.org/10.1093/femsyr/fov068

Marques, W. L., Mans, R., Marella, E. R., Cordeiro, R. L., Van Den Broek, M., Daran, J.-M. G., Pronk, J. T., Gombert, A. K., & Van Maris, A. J. A. (2017). Elimination of sucrose transport and hydrolysis in Saccharomyces cerevisiae: A platform strain for engineering sucrose metabolism. *FEMS Yeast Research*, *17*(1). https://doi.org/10.1093/femsyr/fox006

Özcan, S., & Johnston, M. (1999). Function and Regulation of Yeast Hexose Transporters. *Microbiology and Molecular Biology Reviews*, *63*(3), 554–569. https://doi.org/10.1128/MMBR.63.3.554-569.1999

Rintala, E., Wiebe, M. G., Tamminen, A., Ruohonen, L., & Penttilä, M. (2008). Transcription of hexose transporters of Saccharomyces cerevisiae is affected by change in oxygen provision. *BMC Microbiology*, *8*(1), 53. https://doi.org/10.1186/1471-2180-8-53

Wieczorke, R., Krampe, S., Weierstall, T., Freidel, K., Hollenberg, C. P., & Boles, E. (1999). Concurrent knock‐out of at least 20 transporter genes is required to block uptake of hexoses in *Saccharomyces cerevisiae*. *FEBS Letters*, *464*(3), 123–128. https://doi.org/10.1016/S0014-5793(99)01698-1

**Declaration of Generative AI and AI-assisted technologies in the writing process of the Supplementary Material 2**

During preparation of the Supplementary Materials for this work, the authors used Claude (Anthropic) to assist with improving English language, grammar, and clarity in the supplementary text, and to draft Python code used for generating results tables from BLAST output. Python code used for sequence extraction was independently written by the authors, with AI assistance limited to minor streamlining (e.g., variable naming, formatting, bug fixes). Selection of candidate genes and verification of all information/results, including reciprocal BLAST confirmation and resolution of ambiguous gene annotations, was performed manually by the authors without the help of AI. All code, results, and text were reviewed and verified by the author(s), who take full responsibility for the accuracy and content of the Supplementary Material.
